# An agent-based modelling framework to study growth mechanisms in *EGFR-L858R* mutant cell alveolar type II cells

**DOI:** 10.1098/rsos.240413

**Published:** 2024-07-17

**Authors:** Helena Coggan, Clare E. Weeden, Philip Pearce, Mohit P. Dalwadi, Alastair Magness, Charles Swanton, Karen M. Page

**Affiliations:** ^1^ Department of Mathematics, University College London, London, UK; ^2^ Cancer Evolution and Genome Instability Laboratory, The Francis Crick Institute, London, UK; ^3^ UCL Institute for the Physics of Living Systems, London, UK; ^4^ Cancer Research UK Lung Cancer Centre of Excellence, University College London Cancer Institute, London, UK; ^5^ Department of Oncology, University College London Hospital, London, UK

**Keywords:** agent-based modelling, lung cancer, tumorigenesis

## Abstract

Mutations in the epidermal growth factor receptor (EGFR) are common in non-small cell lung cancer (NSCLC), particularly in never-smoker patients. However, these mutations are not always carcinogenic, and have recently been reported in histologically normal lung tissue from patients with and without lung cancer. To investigate the outcome of EGFR mutation in healthy lung stem cells, we grow murine alveolar type II organoids monoclonally in a three-dimensional Matrigel. Our experiments show that the *EGFR-L858R* mutation induces a change in organoid structure: mutated organoids display more ‘budding’, in comparison with non-mutant controls, which are nearly spherical. We perform on-lattice computational simulations, which suggest that this can be explained by the concentration of division among a small number of cells on the surface of the mutated organoids. We are currently unable to distinguish the cell-based mechanisms that lead to this spatial heterogeneity in growth, but suggest a number of future experiments which could be used to do so. We suggest that the likelihood of L858R-fuelled tumorigenesis is affected by whether the mutation arises in a spatial environment that allows the development of these surface protrusions. These data may have implications for cancer prevention strategies and for understanding NSCLC progression.

## 1. Introduction

Lung cancer is the leading cause of cancer death worldwide [1]. Approximately a quarter of lung cancer patients are ‘never-smokers’ (classified as those who have smoked fewer than 100 cigarettes in their lifetime) [2], and this proportion appears to be increasing [3]. Never-smoking lung cancer is also more common in women than men [4].

Lung adenocarcinomas are thought to arise from alveolar type II (AT2) cells within the healthy alveolar epithelium [5]. The causes of lung cancer in never-smokers are unclear, although previous studies have highlighted germline genetics [6] and exposure to external factors such as infections and radon [7], as well as ambient air pollution [8]. Some specific lung cancer-associated mutations, in particular, in epidermal growth factor receptor (EGFR), are known to occur more frequently among patients with no history of smoking [9], and are found as clonal driver mutations in lung adenocarcinomas [10]. However, their presence alone is insufficient for tumorigenesis: Hill and colleagues have recently reported that 18% of normal lung tissue samples in patients both with and without lung cancer were found to carry EGFR mutations [8]. In order to determine the conditions necessary for lung cancer initiation, we must look further, to the cellular and environmental context in which carcinogenic mutations arise.

It is well-known that the emergence of cancer is probabilistic, and that tumours can take a variety of winding evolutionary paths to acquiring their characteristic hallmarks [11]. No mutation is guaranteed to cause cancer. The number of cells in the human body is such that every possible genetic mutation is likely to exist in at least one cell in everyone [12]; the fact that some people do not develop cancer suggests that not all of these mutations lead to disease in every case. The reproductive fitness of a mutant cell depends not just on tissue context [13] but also on spatial constraints [14] and the ecology of the wider cell population [15]. Subclonal cells interact with each other and compete for resources, giving rise to highly genotypically heterogeneous tumours [16,17]. Within mathematics, the field of evolutionary game theory describes the effect of such interactions on population composition and has seen a variety of recent applications to cancer modelling [18]. However, to make full use of these theories we need strategic quantification: in order to predict a phenotype’s success, we must determine how it interacts with other phenotypes, and thus how its population will rise and fall within a tumour population. To identify the evolutionary benefit of a particular mutation arising in a specific cell type, then, we must consider the effect on that cell’s behaviour. Only then we will be able to predict its contribution to tumorigenesis.

The aim of this study is to analyse the phenotypic changes conferred by the *EGFR-L858R* mutation on AT2 cells. The single-nucleotide substitution L858R in exon 21 comprises around 40% of all EGFR mutations in lung cancer patients [19]. It is a missense mutation, affecting the intracellular kinase domain of the EGFR, which in turn affects a cell’s response to many common growth factors. Biochemical and structural studies have shown that the *EGFR-L858R* mutation promotes dimerization [20] and destabilizes the inactive formation [21] of EGFR, and thus causes abnormal levels of receptor activity. The function of this mutation is also highly clinically relevant. *EGFR-L858R* mutations confer sensitivity to treatment with EGFR tyrosine kinase inhibitors, although acquired resistance can rapidly develop [22,23]. In addition, L858R has been shown to enhance invasiveness in adenocarcinomas [24], and mice with L858R mutations induced in AT2 cells rapidly develop diffuse carcinomas [25]. It is this particular aspect of mutant behaviour that we address in the present study.

We infer an invasive mutant phenotype using an agent-based model (ABM) of organoid growth in a three-dimensional organoid culture, by comparing the structures of spheroids grown monoclonally from mutant and wild-type AT2 cells. ABMs have been common throughout systems biology for many years [26], and are useful for describing systems where individual ‘agents’ (here AT2 cells) interact and reproduce probabilistically according to defined rules. Sometimes also called ‘cellular automata models’, ABMs have been used for almost two decades to construct simulations of tumour growth, in which cells are considered as discrete agents making ‘cell-fate decisions’ in response to changing environmental conditions, such as oxygen depletion [27].

Our focus here is on the emergence of a characteristic ‘budding’ structure in some mutant cell clusters, as opposed to their wild-type counterparts, which grow spherically. These organoids are composed of thousands or tens of thousands of cells and are smaller than is required for the development of vascularity. The field of mathematical modelling of avascular tumour growth is larger than can be fully summarized here (see [28,29] for an introduction). One common methodology is a ‘continuum’ approach, where cell density is described as a continuous scalar field, which evolves with time. Cell–cell interactions and surface-dominated growth can be included in a continuum model [30,31], and the resulting approach is amenable to mathematical analysis, which has shown surface-dominated growth to drive the formation of non-spherical structures. These models are particularly advantageous in the study of reaction–diffusion systems involving nutrient gradients which evolve with time [32], and when analysing the contribution of various biomechanical processes to general morphological instabilities [33]. While these approaches are broadly useful, in many situations the problem we are modelling is three-dimensional, extremely asymmetric and involves fully developed and morphologically specific protrusions, which require high spatial granularity to model. Here, where we wish to test nonlinear growth laws where the discrete number of neighbours a cell has is important, and where the number of cells involved is relatively small (on the order of thousands or tens of thousands), it is both conceptually and computationally easier to model the location and action of each cell directly.

While ABMs have recently been used to shed light on increasingly complex systems of cell–cell interaction [34–37], ABMs built specifically to model organoid growth are relatively rare [38,39]. Most of the existing literature has focused on two systems: the growth of intestinal cells into *in vivo* structures resembling colorectal crypts [40,41], and the morphogenesis of ‘optical cups’, which involves the bending of hollow, initially spherical epithelial layers into characteristic concave structures [42,43]. Such models consider the detailed biomechanical interactions of tens or hundreds of cells, often using off-lattice Voronoi networks, which represent cell centres as point masses that interact using spring forces. These allow the consideration of complex spatial dynamics, such as the work of Thalheim *et al.* [44], who modelled a feedback loop between cell differentiation and the curvature of the resulting organoid to capture the influence of Notch-Wnt signalling on the development of crypt-like intestinal cell organoids. However, such models are computationally expensive and struggle to scale to systems such as ours, involving solid organoids of several thousand cells. ABM frameworks designed to model organoids on this scale [45,46] often require some form of coarse-graining and so are unsuitable for representing short-range interactions between individual cells. To balance this trade-off between scale and detail, we decided to build our own custom model, which allows cell–cell interactions at both short and long ranges, and includes an on-lattice representation of cell movement and extrusion. This model is very cheap to run at scale (i.e. can simulate an organoid in roughly 1 min on a standard laptop), and allows sweeps of our parameters of interest.

In our algorithm, in which space is divided into a fixed three-dimensional lattice, whose points may be either occupied or empty, to simulate cells as they divide and push each other aside. This approach significantly speeds up computation times compared with off-lattice simulation (where the distance between each pair of cells must be recalculated at each time point), which allows for thorough hypothesis testing and parameter sweeps. Our study is driven by the principles of mathematical modelling, wherein complex biological processes are abstracted into high-level mathematical functions. Here, we consider functions which take the number of neighbours a cell has and outputs the probability that cell will divide in a given time step. Whether or not that division actually occurs is decided stochastically, and over time this results in a simulated organoid with an observable morphology. We can alter the forms and parameters of these functions to represent different hypotheses concerning cell growth. The values of these parameters may not correspond to specific biological quantities, but by altering these values—how many neighbours a cell must have before its probability of division begins to decline, for example—we can change the hypothetical rules for cell growth which apply to our system. We can then determine which set of models and parameter regions results in the correct morphologies, and infer the validity of the corresponding rules, without needing to know the biological mechanisms by which they are enforced.

We test various growth hypotheses, including those where cell division is limited by differentiation and by the presence of surrounding cells, and compare the simulated organoids with experimentally observed morphologies. We find that the structural difference between mutant and non-mutant organoids can be explained by four possible hypotheses, all of which result in the concentration of growth among a small number of surface cells. In the first model (Hypothesis A), this ‘surface-localization’ arises from very high levels of differentiation among all cells, combined with more efficient use of available nutrients by mutant cells. In the second model (Hypothesis B), ‘budding structures’ arise from long-range inhibition of cell division by their neighbours, combined with an increase in division rate by mutant cells. In the third model (Hypothesis C), the mutation induces short-range inhibition of neighbouring cells through mechanotransduction, allowing the formation of surface protrusions without any assumed increase in cell fitness. In the fourth model (Hypothesis D), this induced inhibition is long range, through either nutrient depletion or the active secretion of some inhibitor. In all hypotheses, cells do not have to be completely surrounded to suffer inhibition, and are stopped from dividing even when half of their surface is in contact with the nutrient-rich Matrigel. The overall effect is to suppress division in all cells except those on the surface, localizing division within invasive protrusions. We hope this will shed light on the origin of invasive *EGFR-L858R-*positive cancers and the mechanisms by which they emerge from healthy tissue.

## 2. Results

### 2.1. Experimental design

**Figure 1.**
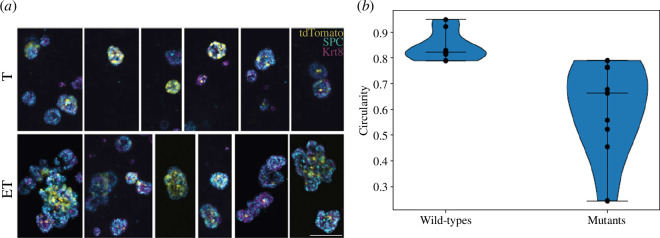
(*a*) Representativethree-dimensional confocal microscopy of AT2 organoids from T mice (top panel) and ET mice (bottom panel). Organoids stained with anti-surfactant protein C (SPC, cyan) and anti-keratin 8 (Krt8, magenta), endogenous tdTomato expression. Results shown from one experiment comprising three mice; *n* = 2 independent experiments. Scale bar represents 100 μm. (*b*) A violin plot of the circularity of observed organoids, with medians indicated. Eight wild-type and nine mutant organoids were captured with sufficient resolution to allow their circularity to be measured. The circularities of the mutant organoids are significantly higher than those of the wild-type (Mann–Whitney *U*-test, p<0.001).

To obtain data for comparison with our hypothesis-testing simulations, we used a genetically engineered mouse model of *EGFR-L858R*-driven lung adenocarcinoma, as described in Hill *et al.* [8] (Rosa26^LSL-tTa/LSL-tdtomato^; *TetO-huEGFR*^L858R^ mice; referred to as ET mice). Here, both the *EGFR-L858R* transgene and tdTomato fluorescent reporter protein are only expressed upon delivery of Cre recombinase. Lox-stop-lox tdTomato mice were used as control (*Rosa26*^LSL-tdTomato/LSL-tdTomato^; referred to as T mice). AT2 cells were purified from non-Cre treated T and ET mice, before inducing expression of the oncogene and/or tdTomato *in vitro* with adenoviral–CMV–Cre incubation using published methods [47,48]. A total of 10 000 AT2 cells were seeded in each organoid assay along with 50 000 supporting lung fibroblasts [49]; in each experiment, only some AT2 cells grew into organoids (with organoid forming efficiency of around 1–2%). After 14 days, organoids were extracted from Matrigel, stained with antibodies and analysed by three-dimensional wholemount confocal microscopy [50]. These analyses revealed a characteristic ‘budding’ structure occurring in mutant organoids, but not wild-type organoids (figure 1). We quantify this effect by analysing the *circularity* of each organoid. An organoid with cross-sectional area a and perimeter p has a circularity $C=\frac{4\pi a}{p^{2}}$. A perfectly circular organoid has $p=2\sqrt{\pi a}$ and so C=1. As an organoid acquires protrusions, its perimeter increases relative to its area, and so its circularity decreases. A shape with infinite perimeter and finite area (i.e. a fractal) has C=0. This is a dimensionless coefficient and so is invariant to the scale at which the organoid is observed. We use the image processing libraries scikit-image [51] and Shapely [52] to automatically find the edges of each organoid and calculate its perimeter and area. This analysis reveals a fairly clean delineation between mutant and non-mutant organoids, with all but one mutant organoid with a circularity C<0.78 (figure 1). Since only mutants have a circularity below this value, we will use this as our criterion for whether a simulated organoid is ‘mutant-like’.

### 2.2. Simulation design

To simulate the development of these structures, we use an on-lattice algorithm. In this framework, three-dimensional space is divided into a 30 × 30 × 30 grid (adjusted to 40 × 40 × 40 where necessary to accommodate an expansive structure). At the end of each simulation step, every lattice point is either occupied by one cell or by none. Each lattice point (except those on the grid’s faces) has 26 Moore neighbours, each of which is considered equally ‘close’ to it within the algorithm. This ensures that when cells are pushed aside by new cells in a simulation step, they are not constrained to move only in six directions (up, down, left, right, forwards or backwards) but can move in 26, allowing the simulation of much more realistic organoid structures. A cell may therefore have up to 26 occupied neighbours. Further model assumptions, and their experimental justification, are summarized in table 1.

**Table 1.** Assumptions made when modelling the system computationally and their experimental justifications.

model assumption	experimental justification
all cells have the same volume (approximately $80\;{µm}^{3}$)	the Matrigel is soft enough that cells are not significantly compressed and may push each other aside when they divide to maintain a constant volume; the figure is extrapolated from measurements of areas of individual cells, approximating cells as spherical
cells remain in contact with their organoid of origin, and do not drift freely through the Matrigel	organoids are cohesive and contiguous; ‘clouds’ of drifting fluorescent cells are not observed
organoids grow independently and there is no cross-talk between them	top-down images of the well suggest that the organoids are seeded very far apart from each other relative to their diameter
external nutrient concentration does not deplete over time	nutrients are regularly replenished by the experimenter
each organoid arises from a single cell	fusion of growing organoids is not experimentally witnessed

The algorithm works as follows. At the start of the simulation, only a single cell is occupied at the centre of the grid. At every time point, each existing cell either divides or does not, according to some probabilistic function of the number of cells in its neighbourhood (which we vary according to the hypothesis being tested). If a cell divides, that lattice point is instantaneously occupied by two cells. This is referred to as the *reproduction* step.

The next step in the algorithm is *spatial adjustment*, where cells on multiply-occupied lattice points are shifted around until they find empty points to settle on, mimicking the physical process of cells pushing each other aside. This step is iterative and repeats until every cell has its own point. At each iteration of the adjustment step, one cell from every multi-occupied point is moved to one of its neighbouring points. The rules for choosing a lattice point to move to are as follows:

— If there are any empty neighbour points, choose between them at random with probability $p\propto e^{\tau n_{1}}$, where $n_{1}$ is the number of occupied lattice points in the *neighbour-point’s* immediate neighbourhood, and τ>0 is a surface tension-like parameter.
— If there are no empty neighbour lattice points, choose one at random with probability $p\propto e^{-\gamma n_{0}}$, where $n_{0}$ is the number of cells currently occupying the candidate lattice point and γ>0 is a ‘repulsion parameter’, such that the focal cell will be less likely to move to lattice points which are already occupied (i.e. have $n_{0}>0$).

The ‘effective surface tension’ parameter τ controls the cohesion of cluster shapes and is designed to mimic the effect of cell–cell adhesion. When it is high, cells will preferentially move to empty points next to occupied points. This is a variable parameter and can be used to qualitatively adjust the amount of surface cohesion in an organoid. The repulsion parameter γ is included to dissuade cells from moving between double-occupied points. Cells are pushed through the cluster to minimize compression, and so will move preferentially to points with fewer cells occupying them. This parameter is designed to mimic the effect of a pressure gradient and is kept high (γ=1).

A key benefit of this algorithm is that whether or not two lattice points are neighbours is only ever calculated once, when the grid is generated, and does not need to be worked out again with every simulation (or, worse, with every simulation step). The number of cells touching a focal cell can be calculated by looking up the list of its neighbouring lattice points and checking whether each of them is occupied. It does not require calculating the distance between the focal cell and every other cell in the organoid. Therefore, its computational cost does not scale with the square of the current number of cells in the cluster, as it would in an off-lattice simulation, but linearly with the number of occupied lattice-points in the system. This means that clusters of many tens of thousands of cells can be simulated in a few seconds, in comparison with equivalent off-lattice simulations, which struggle to simulate more than a few hundred cells efficiently. This computational efficiency allows for quick parameter sweeps.

Clusters are simulated until they reach a certain experimentally realistic physical size, comparable to the observed organoid sizes (maximum diameter of roughly 100 μm, which generally requires around 5000 or 10 000 cells, depending on the division-probability function under investigation). The structure of the resulting organoid is examined and compared with experimental observations.

We use this simulation framework to investigate two broad classes of models concerning organoid growth. In the first class, we assume that the probability that a cell will divide depends only on the number of cells in its immediate neighbourhood. This model is motivated by noting that the Matrigel is highly diffusive (e.g. VEGF has a diffusion coefficient in it of the order of 10^6^ μm^2^ h^−1^ [53]), and so concentration gradients in nutrients and growth factors are difficult to maintain on the timescale of cell division. Cells suppress or induce each other’s division only when their surfaces touch.

In the second model class, we relax this assumption. We suppose instead that a ‘depletion field’ exists around each cell, reducing the ability of nearby cells to divide. In this class, the influence of one cell on another decreases with distance but never vanishes entirely, such that non-neighbouring cells can suppress each other’s growth through either direct inhibitory signalling or competition for resources.

Under both classes of model (i.e. assuming either short- or long-range interactions), mutant-like organoids are created when this mutual inhibition is strong enough to concentrate growth among a small number of surface cells, allowing the development of invasive protrusions. These allow mutant organoids to reach a larger maximum radius using fewer cells than non-mutant organoids, resulting in a highly efficient mode of spatial expansion and suppressing the growth of competing cells.

These results are discussed in more detail below. All parameters used in our model are laid out and defined in table 2. A full list of all model classes and hypotheses considered in this study can be found in table 3.

**Table 2.** The parameters used in this study, their definitions and their values, if fixed.

symbol	parameter	units	fixed or variable
α	a cell’s baseline probability of division in a time step	days^−1^	variable
β	size of the increase in division probability that a cell receives, depending on how many cells are in its immediate vicinity	days^−1^	variable
h	steepness of saturation of the increase in division probability that a cell receives, depending on how many cells are in its immediate vicinity	dimensionless	variable
c	threshold of saturation of the increase in division probability that a cell receives, depending on how many cells are in its immediate vicinity	dimensionless, expressed in number of occupied neighbouring lattice points	variable
S	number of neighbouring lattice points	dimensionless	fixed, S=26
κ	threshold of saturation of the increase in division probability that a cell receives, depending on how many cells are in its immediate vicinity	dimensionless, expressed as a fraction of a cell’s neighbouring lattice points that are occupied	defined as c/S and so varies as c
δt	size of the time steps used in the simulation	days	fixed, δt=0.01
τ	surface tension or cell–cell adhesion	dimensionless	variable
q	the probability that a cell will be born ‘inactive’, i.e. differentiated and unable to reproduce	dimensionless	variable
σ	the probability per unit of time that a cell will become ‘inactive’ as a result of being surrounded by other cells	days^−1^	variable
γ	repulsion from occupied lattice points felt by cells being pushed through the structure	dimensionless	fixed, γ=1

**Table 3.** A description of the candidate hypotheses considered in this study. Each candidate hypothesis belongs to a model class, which assumes that cells interact at either long or short range. We describe the rationale behind each hypothesis, and whether it is able to produce mutant-like organoids (and thus becomes a ‘possible’ hypothesis).

**model class**	**candidate hypothesis number**	**description**	**can produce mutant-like organoids?**
short-range interaction	1	all cells can divide, but nutrient absorption boosts the division rate of isolated cells	no
short-range interaction	2	cells are better able to divide when surrounded by other cells owing to matrix adhesion	no
short-range interaction	3	cells are likely to differentiate at birth, and those cells which can divide are strongly dependent on nutrient absorption	yes (becomes Hypothesis A)
short-range interactions	4	cells inhibit the division of their neighbours, such that cells with a certain number of neighbours are unable to divide	yes (becomes Hypothesis C)
short-range interactions	5	cells suppress each other’s division through the accumulation of effects sustained over prolonged contact	no
long-range interactions	6	cells absorb nutrients necessary for each other’s division, and so inhibit each other’s division through long-range competition	yes (becomes Hypothesis B)
long-range interactions	7	cells secrete inhibitory factors which suppress each other’s division at long range	yes (becomes Hypothesis D)

### 2.3. Nutrient absorption or anchorage-dependent growth alone is insufficient to produce mutant-like organoid structures

#### 2.3.1. Absorption-based growth

A plausible hypothesis for the emergence of ‘budding’ structures is that cells on the surface of the organoid (i.e. those with fewer neighbouring cells) have better access to nutrients and so have a higher probability of division. We hypothesize here that all cells have sufficient access to nutrients to maintain a non-zero division rate even when completely surrounded (see table 1); later we will investigate a regime in which surrounded cells are modelled as having a negligible division rate. This model corresponds to candidate hypothesis 1 in table 3.

**Figure 2.**
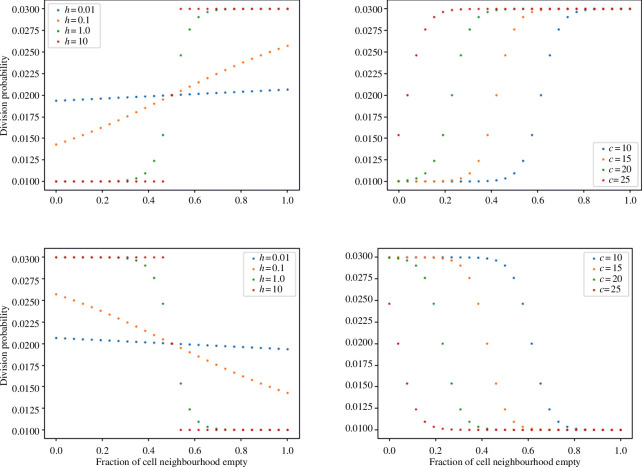
An illustration of the variation in the fitness function $p_{abs}$ (top) and $p_{anc}$ (bottom) with α=1,β=2, i.e. the probability of reproducing in a time interval δt=0.01 (days) under each hypothesis. On the left, c=13 empty neighbours and the steepness h is varied. On the right, h=1 and c=κS is varied.

We test a division probability which is a monotonically increasing function of the number of empty lattice points in a cell’s immediate environment, n, which eventually saturates on physical grounds [54]. Within this framework, the probability p that a cell will divide in a given time step δt is


$$p_{abs}\left(\alpha ,\beta ,h,\kappa ,S,n,\delta t\right)=\left(\alpha +\frac{\beta }{1+e^{-h\left(\kappa -\left(1-n/S\right)\right)}}\right)\delta t,$$

where α>0 is a ‘baseline’ division rate possessed by completely surrounded cells; β is the strength of the nutrient-derived division rate boost (such that the maximum possible division rate of an isolated cell is α+β); κ is the neighbour-occupation threshold, expressed as the fraction of all neighbours, at which the proliferative capacity of a cell decreases; h is the ‘steepness’ of that saturation; and S is the neighbourhood size. The variation of this fitness function with h and κ is illustrated in the top row of figure 2. We will refer to cells with a comparatively high probability of dividing in a given time step as ‘fitter’ throughout this work, and the reader should understand ‘fitness’ here to refer to proliferative capacity, as we do not model cell death.

**Figure 3.**
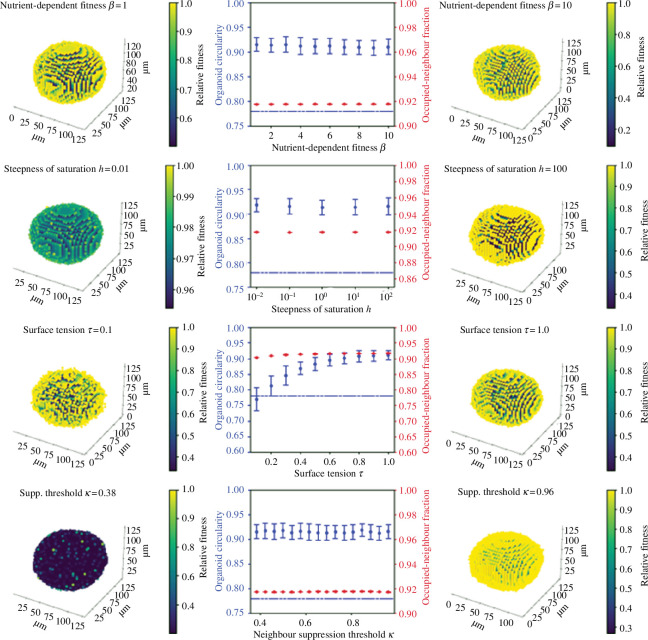
Illustrations of the effect of varying (top row) ‘boost’ β, (second row) steepness h, (third row) cell–cell adhesion τ and (fourth row) fractional threshold κ. Default parameters are β=2,c=20 (κ=0.77), h=1,τ=1. Each graph shows organoid circularity (blue) and the average fraction of an organoid cell’s neighbourhood lattice points which are occupied (red); a completely surrounded cell has occupied-neighbour fraction 1 and an isolated one has an occupied-neighbour fraction 0. These measurements are averaged over 10 simulated organoids and (in the case of circularity) nine different viewpoint angles of each organoid; standard deviations are calculated across all 90 resulting observations, to take account of the variation produced by both underlying structure and viewing angle. Error bars show standard deviations, computed across all observations. The circularity threshold below which an organoid is ‘mutant-like’, C=0.78, is indicated with the dotted blue line. Besides each graph, illustrations of the effects of extreme values on organoid structure are included. Here, the colour of each cell indicates its relative reproductive fitness, scaled to the fittest cell. Each simulation is run until the first time step in which the cluster contains more than 10 000 cells. All axes are in µm; here 10 000 cells give an experimentally realistic diameter of 100 μm.

Throughout these experiments δt=0.01 days, or just under 15 min, to ensure the number of cells generated in any given step is not too high. In figure 3, for example, we see that even completely isolated cells only ever have a 3% probability of reproducing per time step. In general, we consider very large values of h to be biologically implausible, as they would imply sudden changes in a cell’s ability to proliferate if it acquires one or two extra neighbours. We therefore generally restrict ourselves to values of h of order 1 or below. In a system, where β=0 and α>0, all cells would have equal ability to divide and the resulting organoid would be necessarily spheroidal. To test whether any other sets of parameters can result in the development of secondary spheroids, we fix α and vary other parameters to test the possible results of the growth mechanism. Only the values of these parameters relative to α matter; increasing or decreasing all of them by some multiplication factor will simply change the timescale of growth without altering the structure.

Some representative visualizations are shown in figure 3. Here, we display the fractional neighbour threshold κ=c/S, instead of the absolute threshold c, for ease of comprehension. We use default parameters of c=20,h=1 when modelling fitness boost to surface cells; since this leaves a cell with fewer than four neighbours (i.e. an occupied-neighbour fraction less than or equal to 0.85) with greater than or equal to 95% of the fitness of an isolated cell; only after that point does fitness begin to decline.

Varying β,c,h relative to α (the baseline division rate, kept at α=1), and altering the cell–cell adhesion parameter τ, results in uniformly spherical growth (with the surface rougher or smoother depending on whether τ is high or low). The physical reason for this is that if all cells have a non-zero baseline probability of division, then division will occur to some degree or another everywhere in the cluster. Any protrusions that form momentarily at the surface will be evened out, as cells from beneath the outer layer divide, producing new cells which are pushed outwards during the adjustment step. Gaps at the surface, corresponding to unoccupied lattice points, will be filled quickly by newly produced cells squeezed out from the organoid. This is true even when the division-probability boost is very large (10 times the baseline) and when surface tension is low; in any structure where dividing cells remain in contact with each other, the number of mostly surrounded cells will be much larger than the number of completely isolated cells. Thus most reproduction will happen within the cluster, so instabilities at the surface will not develop and the organoid will remain spherical. In all cases, circularity remains above the mutant/wild-type criterion of C=0.78, except for very low τ values, where low cell–cell adhesion produces very rough surfaces.

#### 2.3.2. Anchorage-dependent growth

**Figure 4.**
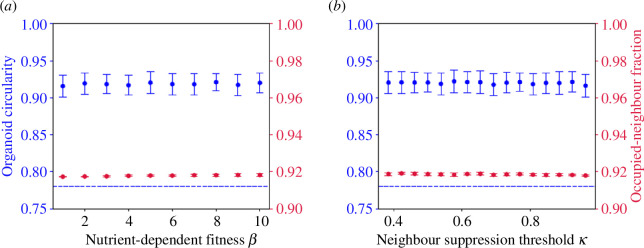
Illustrations of the effect of varying (*a*) ‘boost’ β and (*b*) fractional threshold κ on organoid structure under a hypothesis of anchorage-dependent growth. Default parameters are β=10,c=20 (κ=0.77),h=1,τ=1. Secondary spheroids are never produced. All annotations are as figure 3.

Another plausible hypothesis is ‘anchorage-dependent growth’ [55], whereby cells require adhesion to extracellular matrix (ECM), and thus contact with other cells, in order to progress through the cell cycle. This suggests that division probability increases monotonically with the number of neighbouring cells (candidate hypothesis 2 in table 3). This motivates a division-probability function of the form


$$p_{anc}\left(\alpha ,\beta ,h,\kappa ,S,n,\delta t\right)=p_{abs}\left(\alpha ,\beta ,-h,\kappa ,S,n,\delta t\right)=\left(\alpha +\frac{\beta }{1+e^{h\left(\kappa -\left(1-n/S\right)\right)}}\right)\delta t,$$

where all parameters have the same meaning as before. The variation of this fitness-function with h and κ is illustrated in the bottom row of figure 2. Now κ is the threshold fraction of the neighbourhood that must be occupied before a cell’s proliferative capacity *increases*. We find that this leads to spherical growth since cells benefit from being as close to one another as possible, with protruding cells at a disadvantage (see figure 4).

### 2.4. High levels of differentiation combined with efficient use of nutrients by mutant cells can result in ‘budding structures’

A further testable hypothesis is differentiation-based growth (candidate hypothesis 3 in table 3). We have previously assumed that all cells in the model are stem cells, and all equally able to divide given the correct conditions. However, stem cells might also give rise to *differentiated* cells, which serve some specialized functions but are not able to divide further. Could differences in the division patterns of progenitor cells create budding structures?

To test this, we make a slight alteration to our framework. We use a division function of the form $p_{abs}$, assuming some fitness boost to surface cells, but further divide cells into two types: ‘active’ (here corresponding to stem cells) and ‘inactive’ (here corresponding to differentiated cells). Both types of cells can be pushed aside when other cells reproduce, but only active cells can divide. When an active cell divides, the new cell is active with probability 1−q and inactive with probability q. We refer to q as the probability of differentiation (as defined in table 2). We also implement a change during the adjustment step, to prevent newly produced cells from ending up very far from their mother cells as soon as they are produced. We keep track of the type of cell last added to any lattice point. When a cell is moved from that point, we make sure to keep at least one cell of that type there after the movement step. The moved cell is chosen at random from those remaining. For example, if a point initially contains one active cell, and has an inactive cell moved to it during the adjustment step (so that it is double-occupied), then in the next step, the active cell will be moved. This ensures that cells are not propagated too far through the cluster during a single adjustment step.

**Figure 5.**
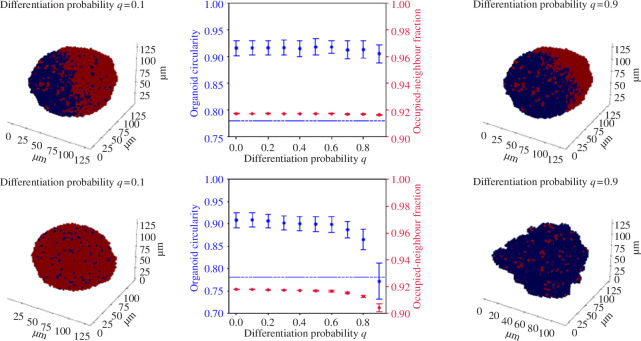
The effect of varying differentiation probability on organoid structure: with (top) β=0 and (bottom) with β=10,c=20(κ=0.77),h=1. In all experiments, τ=1.0,α=1. All experiments are run to 10 000 cells. Here, active cells are shown in red, and inactive cells in blue.

We see that high levels of differentiation enhance the structural effects of absorption-dependent fitness to produce ‘bubbling growth’ (see figure 5). The combination of these two mechanisms is sufficient to limit division to a small number of cells almost entirely located on the surface. In the absence of absorption-dependent fitness, active cells are distributed throughout the body of the organoid (as no benefit accrues to being located on the surface), but tend to be skewed towards one side of the organoid or the other; as only active cells can produce other active cells, any chance bias in their location will reinforce itself once it has arisen.

In §2.5, we consider other model systems which can produce organoids below the ‘mutant-like’ circularity threshold. While they differ in their physical motivation, all produce this same surface-localization effect.

### 2.5. Strong neighbour suppression is sufficient to produce mutant organoid structures

The simulations above establish that so long as surrounded cells are able to reproduce, clusters will remain largely spherical. What happens if surrounded cells are completely prevented from dividing through means other than differentiation? To test this hypothesis, we use the division-probability function


$$p_{\text{NS}}\left(n,\delta t\right)=p_{abs}\left(0,\beta ,h,\kappa ,S,n,\delta t\right)=\frac{\beta \delta t}{1+e^{-h\left(\kappa -\left(1-n/S\right)\right)}},$$

which is a special case of $p_{abs}\left(n,\delta t\right)$ with α=0. Here, cells must have empty neighbour-points in order to reproduce; the baseline division probability of surrounded cells is almost zero. (This corresponds to candidate hypothesis 4 in table 3.) This particular dependency may arise for a couple of mechanistic reasons. If cells are sufficiently densely packed that nutrients cannot reach cells at the centre of the cluster, we might expect them to stop dividing. This would only happen when cells are completely surrounded, however, and so would correspond to a high threshold κ. If cells were stopped from dividing when not completely surrounded, however, we might attribute this to active inhibition by neighbouring cells.

**Figure 6.**
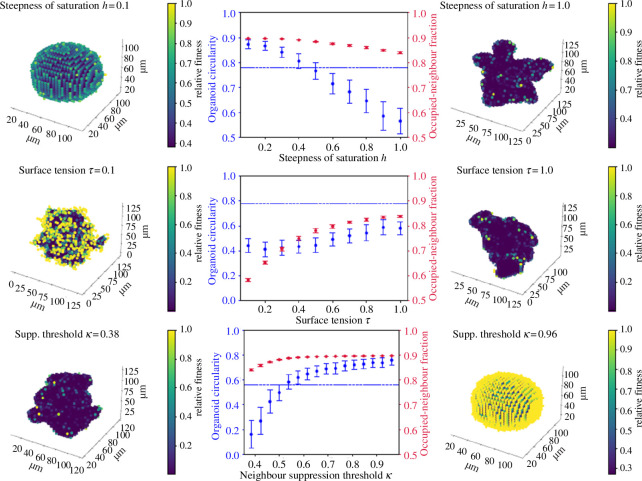
Illustrations of the effect of varying (first row) steepness h, (second row) cell–cell adhesion τ and (third row) threshold κ. For all simulations α=0,β=10. Default parameters are κ=0.38,h=1,τ=1; all parameters are at these values unless stated otherwise above a graph. All axes are in μm; here 5000 cells give an experimentally realistic diameter of 100 μm. Colour indications are as before.

Using this growth mechanism, sustained protrusions can develop, and we observe structures which look remarkably similar to those shown in experiments (figure 6). Mutant-like circularity is established at a threshold of c=13 or κ=0.5 and a saturation steepness of h=1, regardless of the level of cell–cell adhesion. This corresponds to a division probability which is saturated for a cell with fewer than 11 neighbours (where 42% of its surface is covered) and close to zero for a cell which has more than 15 (where 58% of its surface is covered). This low threshold is worth noting: cells that are still very much in contact with the nutrient-rich Matrigel cannot divide. These extremely high levels of neighbour inhibition suggest either that local competition for nutrients is so strong that even surface cells can be deprived of resources by their neighbours, or else that mutant cells actively inhibit each other’s growth through mechanotransduction. Both explanations take the same mathematical form and so are permitted by this model.

Decreasing the steepness h means that the benefit of neighbouring empty lattice points (i.e. the inhibitory effect of neighbour–neighbour signalling) becomes approximately linear, which allows surrounded cells to divide, leading to spherical growth. In order to generate secondary spheroids, the benefit to surface cells must be steeply nonlinear, so the behaviour of these cells is a ‘switchlike’ function of their circumstances. Surface-dependent growth requires the ‘surface’ to be distinctly and sharply advantaged, i.e. for the proliferative capacity of a cell to increase significantly when it has below a certain number of neighbours.

Cross-sectional analysis of the simulation shows that these organoids grow as solid structures, without ‘air pockets’, which also matches experimental observation (when grown organoids are removed from the Matrigel and analysed at the end of the experiment, they are found to be solid).

**Figure 7.**
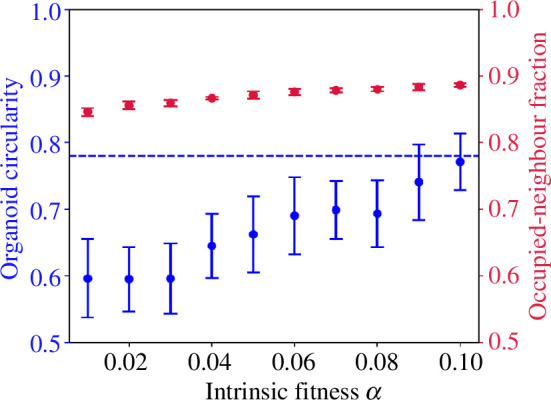
Illustration of the effect of varying inherent fitness α in organoids grown with β=10,τ=1.0,h=1,c=10.

We can also see that the baseline division rate of surrounded cells, α, must be very small compared with β=10 to allow the development of budding growth from figure 7, which shows the emergence of mutant-like organoids as α is decreased from 0.1 (near-spheroidal growth) to zero (budding growth). In order to stay beneath the mutant-like organoid threshold C=0.78, the baseline division rate of the cells must be negligible (i.e. α<0.01β).

We make two further significant observations. Firstly, when neighbour suppression is high, the experimentally witnessed diameter of about 100 µm can be replicated with only 5000 cells (versus roughly 10 000 without suppression; see §2.4). Intuitively, this is the effect of protrusion-based growth: organoids with high neighbour suppression limit their division to surface cells and so grow outwards, as opposed to clustering together at the centre. In other words, neighbour suppression results in more invasive organoid behaviour. This could partly explain why carcinomas induced in mice with this mutation are so diffusive [25]: neighbour suppression would force cells to grow away from each other, and a mutant cell at the boundary of an expanding clone would both be able to prevent the division of its wild-type neighbours and be sharply advantaged compared with a mutant cell at the organoid’s centre.

#### 2.5.1. Accumulation of suppression produces deformations but not mutant-like ‘budding’

**Figure 8.**
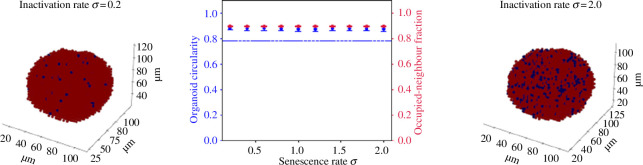
The effect of inactivation rate on ‘accumulated inactivity’-driven growth as described above. Here, α=10,h=τ=1,κ=0.38 and σ is varied. All simulated organoids are run to 5000 cells. Active cells are shown in red and inactive cells in blue.

The picture that emerges is now clearer. Our simulations suggest that cells in budding organoids are much fitter than cells in spheroidal organoids (represented by low β in our model), but surrounded cells are prevented from reproducing by contact with neighbouring cells (represented by a negligibly low baseline α). Possible mechanisms for this suppression are discussed in §3. One important note is that the mechanism of suppression does not necessarily have to be exclusively current; cells which remember being surrounded can also produce deformations. To illustrate this, we can consider an alternative model, similar to the differentiation model in candidate hypothesis 3, in which all cells are born active and become inactive in response to being surrounded (candidate hypothesis 5 in table 3). In this model, all active cells have the same per-time-step division probability $p_{\text{diff}}(n,\delta t)=\alpha \delta t$. However, in a given time step, an active cell with n empty neighbouring lattice points has a probability $\frac{\sigma \delta t}{1+e^{h(\kappa -(1-n/S))}}$ of becoming inactive, for some ‘inactivation rate’ σ. This probability is almost zero when the cell is isolated and maximal when it is surrounded. A cell which is surrounded for a long time may initially be active, but is very likely to eventually be forced into inactivity through prolonged exposure to other cells. Even if it is later pushed to the surface, it will remain inactive. We call this mechanism ‘suppression accumulation’. The results of simulations with similar ‘inhibition strength’ parameters α=10,κ=0.38,h=1,τ=1.0 and various values of σ are shown in figure 8. (We use a high value of α here for computational efficiency, since continual inactivation will considerably increase the timescale of organoid growth without any corresponding increase in division rate.) For high rates of inactivation, we observe non-spherical deformations, enough to bring the circularity below the threshold C=0.78 required to classify an organoid as ‘mutant-like’ in some cases. However, visual inspection shows that these structures are in fact ellipsoidal, not budding. As with differentiation-based growth, the fact that active cells are only produced by other active cells means that proliferation can easily become lopsided; here small groups of active cells proliferate from an off-centre point within the cluster, resulting in an elongated ‘rugby-ball’ shape. As cells beneath the surface are still able to reproduce, however, we do not see the development of protrusions. We conclude that, while cumulative suppression may exist within the structure, it is insufficient to produce truly ‘mutant-like’ organoids.

### 2.6. Long-range neighbour suppression is sufficient to produce budding structures

Finally, we relax the assumption that cells can only interact when their surfaces touch, and consider a model in which cells can interact at larger distances. This can happen because cells absorb nutrients within the Matrigel, depleting the resources available to another cell further away (candidate hypothesis 6 in table 3); or because cells actively secrete some inhibitory factor, whose concentration decreases with distance (candidate hypothesis 7 in table 3). Both hypotheses result in mathematically equivalent models. In the following explanation, we will discuss this phenomenon in terms of active inhibition. We assume that cell i at position ${\underline{x}}_{i}$ secretes some factor with concentration $\rho_{i}(\underline{x},t)$ that inhibits the growth of other cells (and that this may simply be ‘the absence of nutrients’). This factor is diffusive and so, outside the cell itself, obeys the diffusion equation (for some diffusion constant D),


$$\frac{\partial \rho_{i}}{\partial t}=D\nabla^{2}\rho_{i}.$$

We assume the nutrient, applied externally by the administrator, diffuses much more rapidly than it decays and so we need not consider a degradation term (which would lead to an exponential solution in one dimension and a Bessel function form in two). We assume that, on the scale of cell reproduction, the concentration of this inhibitory factor has reached a steady state and further that it is radially symmetric about the cell, so that at any point which is a distance $r_{i}$ from the centre of cell i (assuming that $r_{i}>r_{0}$, the cell radius) the inhibitory factor obeys the equation


$$\nabla^{2}\rho_{i}=\frac{1}{r_{i}^{2}}\frac{\partial }{\partial r_{i}}\left(r_{i}^{2}\frac{\partial \rho_{i}}{\partial r_{i}}\right)=0.$$

We assume that $\rho_{i}\to 0$ as $r_{i}\to \infty$, i.e. that the influence of one cell on another vanishes at infinite separation. This leads to $\rho_{i}=\frac{\lambda }{r_{i}}$ for some constant λ, which we take the same for all cells within the same organoid. We further assume that cells act as point sources of this factor, that cells can be approximated by spheres and absorb this factor at their surfaces (where concentration is finite). We need only calculate intercellular influence, as the effect of each cell’s secretions on its own division rate is absorbed into the baseline fitness. When calculating the concentration of inhibitory factor secreted by a distant cell at the surface of a focal cell, we neglect any variation in concentration across this surface and assume that the concentration is dependent only on the distance between the two cell centres. This leads to a fitness $\alpha -\mu \Sigma_{j\neq i}\rho_{j}({\underline{x}}_{i})$ for cell i, where $\rho_{j}({\underline{x}}_{i})$ denotes the concentration of the inhibitory factor secreted by cell j at the position of cell i, and μ is some scaling factor representing the effect of this substance on cell division rates. Adding this together, the probability that cell i will divide becomes


$$p_{\text{LR}}\left(\delta t\right)=\alpha \left(1-\eta \Sigma_{j\neq i}\frac{1}{r_{ij}}\right)\delta t,$$

where $r_{ij}$ indicates the distance between the centres of cell i and cell j, and for some scaling factor η which we vary, and which we refer to as the ‘inhibition level’. We take η small enough that this is always positive in the two-cell case (i.e. to keep individual influences small); within the simulation, this probability may drop below zero for a highly surrounded cell in the case of strong depletion, in which case the cell simply cannot divide (i.e. has an effective $p_{\text{LR}}=0$).

**Figure 9.**
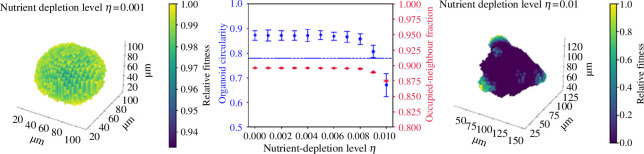
The effect of long-range inhibition on organoid growth. Here, α=1,τ=1 and η is varied. All simulated organoids are run to 5000 cells. Colour indicates the relative fitness of a cell, i.e. its probability of division relative to the most fit cell in the organoid, to visually highlight where in the organoid division occurs.

**Figure 10.**
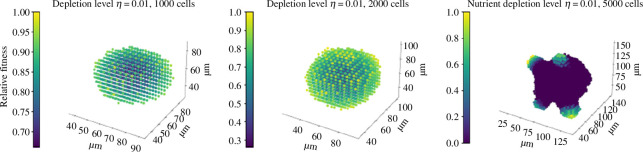
The effect of long-range inhibition on organoid growth. Here, α=1,τ=1,η=0.01 and final size is varied. Colour indicates the relative fitness of a cell.

Figure 9 shows the effect of increasing this long-range inhibition. At η≈0.01, long-range depletion is sufficient to create mutant-like organoids. As previously, this budding structure occurs because all but a small number of cells located in surface protrusions are prevented from dividing, even those in contact with the Matrigel. Figure 10 shows the dependence of this structure on organoid size (though this cannot be assessed quantitatively, as our image processing pipeline is unable to accurately define a perimeter for small organoids, where the scale of the structure is close enough to the scale of individual cells that the protrusions of individual cells interfere with the determination of the outline). Thus, the distinction between mutant and non-mutant organoids can be explained either by universal long-range depletion and by an increase in division rate among mutant organoids, such that only mutants reach a number of cells required to prompt the emergence of budding structures or by an increase in long-range suppression by mutant cells, either through active inhibitory signalling or enhanced absorption of key nutrients. This latter possibility does not preclude an increase in division rate, but does not require it. We are unable to determine the number of cells included in a non-spherical structure through cross-sectional areas, so it is unclear from our data whether mutant organoids necessarily contain more cells than non-mutants. Further experiments should weigh all organoids to clarify this question.

While higher inhibition should decrease the overall amount of division in the organoid, it may not necessarily decrease the observed diameter, as the development of surface protrusions packs cells very inefficiently and allows rapid expansion into the surrounding Matrigel. In general, we can conclude that high levels of long-range inhibition are sufficient to concentrate division within surface protrusions and result in bubbling growth.

## 3. Discussion and conclusion

In this study, we have developed an ABM approach to simulate the growth of mutant and non-mutant organoids. We have found that the secondary spheroids formed by mutant clusters can be explained by several hypotheses, all of which result in the restriction of division to a small number of surface cells in order to facilitate protrusion. This study builds upon the body of theoretical and computational work establishing the existence of instabilities in organoid growth [31–33], and our ABM approach allows us to straightforwardly calculate the detailed morphology of the resulting developed protrusions.

We find that four possible models can predict the emergence of mutant-like organoids. In one (Hypothesis A), high levels of differentiation exist in all organoids, but mutant cells make more efficient use of available resources. In another (Hypothesis B), long-range inhibition of cell division allows budding structures to develop in mutant organoids but not non-mutants because of an increase in inherent division rate. In the third and fourth, the mutation induces either short- (Hypothesis C) or long-range (Hypothesis D) inhibition of division between neighbouring cells.

While we are unable to distinguish between these mechanisms at present, we can suggest the following set of experiments which could be used to do so. To assess Hypothesis A, flow cytometry can be used to determine the levels of differentiation across all organoids, and the relationship between nutrient levels and division rate should be measured in both mutant and non-mutant cells. This hypothesis predicts that all cells should be highly differentiated and that the proliferation of mutant cells should be much more sensitive than non-mutants to differences in nutrient concentration.

Hypotheses B–D all predict that cells can inhibit each other’s division but are agnostic as to whether this is active (through the secretion of some inhibitor) or passive (through nutrient depletion). Hypothesis B predicts that mutant and non-mutant cells should be equally sensitive to nutrient depletion (and that mutant cells should have a generally higher division rate). This dependence might be strong (which suggests passive inhibition) or negligible (which suggests that inhibition is active, or at least independent of any nutrients supplied by the experimenter). Hypotheses C and D suggest that intercell influences are *induced* by the mutation, which could be detected by co-culturing mutant and non-mutant cells and measuring their division rate with time-lapse video. If this inhibition is short range (Hypothesis C), then division should be inhibited only for those cells directly in contact with mutant cells. If it is long range (Hypothesis D), division will be suppressed for all non-mutant cells co-cultured with mutant cells in comparison with those cultured alone. In general, we will be able to detect active inhibition if the division rate of a cell can be lowered by contact or co-culture with other cells, but not by an experimenter-induced decrease in nutrient levels. A summary of the expected results of each experiment needed to confirm each hypothesis is provided in table 4.

**Table 4.** Suggested experiments and their expected results under each hypothesis. Note that a ‘yes’ here means that a positive result is *necessary* to confirm each hypothesis (though not always sufficient); a ‘no’ means merely that the relevant hypothesis does not necessarily predict it.

prediction	Hypothesis A	Hypothesis B	Hypothesis C	Hypothesis D
flow cytometry experiments should show mutant cells are more commonly differentiated than wild-type cells	yes	no	no	no
depletion experiments should show the division rates of mutant cells are more sensitive to nutrient levels than wild-type cells	yes	no	no	no
depletion experiments should show the division rate of mutant cells is higher at the same nutrient level than wild-type cells	no	yes	no	no
co-culture experiments should show that cells co-cultured with mutant cells are less proliferative than those co-cultured with wild-type cells	no	yes	no	yes
co-culture experiments should show that cells in direct contact with mutant cells are less proliferative than those in direct contact with wild-type cells	no	yes	yes	yes

The mechanism of this active inhibition, if present, is currently unclear. Contact-inhibited proliferation, the phenomenon whereby cells stop dividing in areas of high local density [56], is known to be mediated by EGF concentration [57] and so might in theory be affected by the presence of an EGFR mutation. Variations in contact inhibition were found to produce morphologically distinct organoid structures in the simulations of Karolak *et al*. [58]. However, contact-inhibition is generally lost during the development of cancer [59], and so it would be surprising if such a cancer-associated mutation as *EGFR-L858R* were found to induce it. Instead, we expect either that mutants compete more for nutrients than non-mutants or that mutant cells secrete an antagonist that halts the division of their immediate neighbours, wild-type or mutant. A similar phenomenon has recently been observed among intestinal stem cells with mutated *Apc* by Flanagan *et al.* [60]. If this is happening in our system, then this should give *EGFR-L858R* mutant AT2 cells a significant competitive advantage over non-mutant cells *in vivo* and when grown in co-culture, and at least partially explain the invasiveness of L858R-mutant carcinomas. Further experimental work is needed to verify this. If a signalling pathway which leads to neighbour suppression could be identified, this might be pharmacologically targetable and have implications for the prevention and treatment of non-small cell lung cancer among never-smokers.

The existence of even passive inter-cell inhibition, if confirmed, would suggest that spatial environment strongly influences whether or not a pre-cancerous cell is able to develop into a lesion. Cell clusters in narrow confines will inhibit each other’s division and stall the overall growth of the cancer. However, given enough room, pre-cancerous cells can escape each other’s influence, allowing the development of invasive protrusions. Our work emphasizes the importance of considering spatial structure and cell–cell interaction in tumorigenesis, and to consider the circumstances of cell growth when determining whether or not a particular mutation will lead to cancer. Only by considering mutant cells within their full context, ecological and otherwise, will we be able to untangle the mechanisms that lead to cancer.

## 4. Material and methods

### 4.1. Animal procedures

Animals were housed in ventilated cages with access to food and water ad libitum. All animal procedures were approved by The Francis Crick Institute Biological Research Facility Strategic Oversight Committee, incorporating the Animal Welfare and Ethical Review Body, conforming with UK Home Office guidelines and regulations under the Animals (Scientific Procedures) Act 1986 including Amendment Regulations 2012. Both male and female animals aged 6–15 weeks were used.

*EGFR*-L858R [Tg(tet-O-EGFR-L858R)56Hev] mice were obtained from the National Cancer Institute Mouse Repository. Rosa26tTA and Rosa26-LSL-tdTomato mice were obtained from Jackson laboratory and back-crossed as previously described by Hill *et al.* and Politi *et al.* [8,25]. After weaning, the mice were genotyped (Transnetyx, Memphis, TN, USA), and placed in groups of 1–5 animals in individually ventilated cages with 12 hours daylight cycle.

### 4.2. Fluorescence-activated cell sorting

For flow cytometry sorting of AT2 cells, minced lung tissue was digested with Liberase TM and TH (Roche Diagnostics) and DNase I (Merck Sigma-Aldrich) in HBSS (Gibco) for 30 min at 37°C in a shaker at 180 r.p.m. Samples were passed through a 100 μm filter, centrifuged (300*g*, 5 min, 4°) and red blood cells were lysed for 5 min on ice using ACK buffer (Life Technologies). Cells were blocked with anti-CD16/32 antibody (BD) for 10 min. Extracellular antibody staining was then performed for 30 min on ice (see table 5), followed by incubation in DAPI (Sigma-Aldrich) to label dead cells. Cell sorting was performed on Influx, Aria Fusion or Aria III machines (BD). AT2 cells were defined as DAPI-CD45-CD31-Ter119-EpCAM+MHC Class II+CD49f- as previously described in Major *et al.* [47].

**Table 5.** The materials used in these experiments.

antigen	fluorochrome	vendor	cat no.	dilution
CD45	BV421	Biolegend	103134	1/150
CD31	BV421	Biolegend	102423	1/150
Ter119	BV421	Biolegend	1162	1/150
EpCAM	APC-Fire750	Biolegend	118230	1/150
MHC Class II	FITC	Biolegend	107606	1/150
CD49f	PE-Cy7	eBiosciences	25-0495-82	1/150

### 4.3. Organoid forming assay

AT2 cells were isolated from control T or ET mice, without *in vivo* Cre induction, incubated *in vitro* with 6 × 10^7^ PFU ml^−1^ of Ad5-CMV-Cre in 100 μl per 100 000 cells three-dimensional organoid media (DMEM/F12 with 10% FBS, 100 U ml^−1^ penicillin–streptomycin, insulin/transferrin/selenium, L-glutamine (all GIBCO) and 1 mM HEPES (in-house)) for 1 hour at 37°C as detailed in Dost *et al.* [48]. Cells were washed three times in PBS, before 10 000 cells were mixed with a murine lung fibroblast cell line (MLg2908, ATCC, 1 : 5 ratio) and resuspended in growth factor reduced Matrigel (Corning) at a ratio of 1 : 1. A total of 100 μl of this mixture was pipetted into a 24-well transwell insert with a 0.4 μm pore (Corning). After incubating for 30 min at 37°C, 500 μl of organoid media was added to the lower chamber and media changed every other day, following previous methods [49]. Bright-field and fluorescent images were acquired after 14 days using an EVOS microscope (Thermo Fisher Scientific) and quantified using FiJi (.2.0.0-rc-69/1.52 r, ImageJ). For wholemount staining of organoids, organoids were prepared according to previous methods [50] and stained with anti-proSPC (Abcam, clone EPR19839) and anti-keratin 8 (DSHB Iowa, clone TROMA-1). Three-dimensional confocal images were acquired using an Olympus FV3000 and analysed in FiJI.

#### 4.3.1. Computational analysis

All organoids captured at sufficient resolution to accurately determine an area and perimeter were analysed using the scikit-image and Shapely libraries. Images were converted to greyscale and passed through a Gaussian filter with σ=0.8, for all except the non-mutant organoid labelled ‘*t*2’, which was captured at lower resolution and required σ=1.0 for the perimeter to be found correctly. Contours were found using the function shapely.measure.find-contours with parameter ‘level’ = 0.5; these were then visually verified. The concave hull of these contours was then computed using shapely.concave-hull with parameter ‘ratio’ = 0.05. Circularity was determined using the variables hull.area and hull.length (to describe the perimeter). The same default parameters were applied to the analysis of all simulated organoids. When calculating the circularity of these organoids, nine different images were taken from different viewpoint angles, using all possible combinations of elevation and azimuthal angles = −60°, −20°, 20°.

## Data Availability

Data and relevant code for this research work are stored in GitHub [61] and have been archived within a Zenodo repository [62]. The EGFR-condition images have been previously published in [8]. All other data are original.
